# The association between positive parent–child interaction and mental health outcomes in children aged 1–11 years in Canada

**DOI:** 10.17269/s41997-025-01073-x

**Published:** 2025-06-17

**Authors:** Peter Yassa, Justin Thielman, Andrea Gonzalez, Mackenzie Martin, Daniel W. Harrington, Sarah Carsley

**Affiliations:** 1 Department of Health Promotion, Chronic Disease, and Injury Prevention, Public Health Ontario, Toronto, ON Canada; 2 Department of Health Research Methods, Evidence, and Impact, McMaster University, Hamilton, ON Canada; 3 Department of Psychiatry and Behavioural Neurosciences, Offord Centre for Child Studies, McMaster University, Hamilton, ON Canada; 4 Dalla Lana School of Public Health, University of Toronto, Toronto, ON Canada

**Keywords:** Parent–child interaction, Mental health, Children, Public health, Epidemiology, Interaction parent-enfant, Santé mentale, Enfants, Santé publique, Épidémiologie

## Abstract

**Objectives:**

The prevalence of mental health disorders in Canada has increased over the past 10 years. Positive parent–child interaction (PCI) is a potential protective factor for child mental health, but has not been explored in a Canadian context. This study aimed to determine the association between positive PCI and mental health outcomes in children ages 1–11 years in Canada.

**Methods:**

Participants were included from the 2019 Canadian Health Survey on Children and Youth (CHSCY) (*N* = 28,874). PCI was assessed using five items, reported by the parents. The combined PCI score was also derived. Parent-reported general child mental health was the main outcome. Multivariable logistic regression models, adjusting for confounders, were performed.

**Results:**

Only 2.8% of parents reported their child’s mental health to be “fair/poor”. The adjusted analysis did not show an association between combined PCI score and parent-reported general child mental health (OR = 0.96; 95%CI 0.91–1.01). Models assessing individual PCI items did not show significant associations with general child mental health, except for the “laughs with child” item.

**Conclusion:**

While it is theorized that positive PCI is predictive of child mental health, this study did not find a consistent association, except for the frequency at which the parent laughs with the child. This suggests that PCI, as measured in CHSCY, is not a strong indicator of child mental health. Yet, as PCI is an important parenting concept, the PCI items in the CHSCY may not adequately capture the intended construct. Future studies should consider assessing the construct validity of these items.

## Résumé

**Objectifs:**

La prévalence des troubles mentaux au Canada a augmenté au cours des dix dernières années. L’interaction parent-enfant (PCI) positive est un facteur de protection potentiel pour la santé mentale des enfants, mais elle n’a pas été étudiée dans un contexte canadien. La présente étude visait à déterminer l’association entre l’interaction parent-enfant positive et les résultats en matière de santé mentale chez les enfants âgés de 1 à 11 ans au Canada.

**Méthodologie:**

Les participants ont été inclus dans l’Enquête canadienne sur la santé des enfants et des jeunes (ECSEJ) de 2019 (N = 28 874). L’interaction parent-enfant a été évaluée à l’aide de cinq éléments, déclarés par les parents. Le score PCI combiné a également été calculé. Le principal résultat était la santé mentale générale des enfants déclarée par les parents. Des modèles de régression logistique multivariable, ajustés pour tenir compte des facteurs de confusion, ont été réalisés.

**Résultats:**

Seuls 2,8% des parents ont déclaré que la santé mentale de leur enfant était « passable/mauvaise». L’analyse ajustée n’a pas révélé d’association entre le score PCI combiné et la santé mentale générale des enfants déclarée par les parents (RC = 0,96; IC à 95%: de 0,91 à 1,01). Les modèles évaluant les éléments individuels de l’interaction parent-enfant n’ont pas révélé d’associations significatives avec la santé mentale générale des enfants, à l’exception de l’élément « rit avec l’enfant».

**Conclusions:**

Bien qu’il soit théorisé qu’une interaction parent-enfant positive permet de prédire la santé mentale des enfants, cette étude n’a pas trouvé d’association cohérente, à l’exception de la fréquence à laquelle le parent rit avec l’enfant. Cela suggère que l’interaction parent-enfant, comme mesurée dans l’ECSEJ, n’est pas un indicateur solide de la santé mentale des enfants. Pourtant, comme l’interaction parent-enfant est un concept parental important, les éléments de l’interaction parent-enfant de l’ECSEJ pourraient ne pas refléter de manière adéquate le concept visé. Les études futures devraient envisager d’évaluer la validité conceptuelle de ces éléments.

## Background

Over the course of their lifetimes, one in five Canadians will experience a mental health disorder (Cosco et al., 2022). The prevalence of mental health disorders in Canada has steadily increased over the past 10 years (Stephenson, 2023). Research has shown that poor mental health in childhood and adolescence tends to predict poor mental health in early adulthood as well as lower quality of life and life satisfaction (Gershon et al., 2013; Schlack et al., 2021). Further, having poor mental health in childhood is associated with multiple health outcomes later in life, including increased morbidity and all-cause mortality (Jokela et al., 2009; Stein et al., 2010). Therefore, exploring how mental health conditions manifest in childhood is essential to reducing their impact later in life.

One potential protective factor for mental health is positive parent–child interaction (PCI) in childhood. PCI refers to the quality with which parents interact with their child, including positively communicating, showing affection, and providing social support (Horstman et al., 2016). PCI can be assessed by asking parents about their interactions with their child, such as how often they focus attention on their child or laugh with their child (Statistics Canada, 2024). Previous research has shown that the quality of PCI in the early years serves as a foundation for child development across multiple domains. Positive PCI activities such as shared reading and verbal engagement enhance literacy and foster cognitive stimulation in children (Dodici et al., 2003; Weisleder et al., 2019). Additionally, parenting behaviours characterized by emotional support and warm acceptance contribute to better emotional regulation and social engagement (Smith et al., 2006). Positive PCI also plays a crucial role in mental health outcomes (Ge et al., 2009; So et al., 2021). In a cross-sectional study in the United States of 113,834 public school students in Minnesota, parent–child connectedness was associated with reduced depressive symptoms and suicide for adolescents in grades 8, 9, and 11 (So et al., 2021). Similarly, in a longitudinal study of 756 adolescents in the United States, parent–child closeness was associated with fewer depressive symptoms 3 years later (Ge et al., 2009).

While multiple studies have focused on specific elements of positive PCI, few have examined positive PCI as a combined score in large population-based studies. In Shan et al. (2019), PCI was assessed using the Chinese Parent–Child Interaction Scale (CPCIS), a standardized scale to measure PCI in China. This retrospective study conducted in a representative sample of 20,324 children ages 3–4 years in Shanghai found that PCI acted as an effect modifier for the effect of maltreatment during childhood on children’s social problems, where high frequency of positive PCI mitigated the effect (Shan et al., 2019). Similarly, Katsantonis and McLellan (2024) assessed PCI using the Parent–Child Relationship Short Form Pianta scale in a community sample of 10,703 children in the United Kingdom. This longitudinal study found that positive PCI, including emotional closeness and reduced conflict, acted as a protective factor for pro-sociality and mental health during adolescence. However, there is a scarcity of studies assessing positive PCI as a combined score within a Canadian population-based context.

Positive parenting is an important topic in public health and is part of the World Health Organization’s guidelines for improving early childhood development (World Health Organization, 2020). However, measuring parenting at the population level presents many challenges, including multiple contextual and cultural considerations (Lindhiem et al., 2019). Therefore, determining the relevance of a combined PCI score from a population-representative Canadian health survey has important implications in tackling mental health issues in Canadian children by guiding policy and program recommendations for positive parenting practices. The objective of this study was to determine if there is an association between positive PCI and parent-reported mental health outcomes in children aged 1–11 years in Canada using the 2019 Canadian Health Survey on Children and Youth (CHSCY).

## Methods

This study was approved by the Ethics Review Board at Public Health Ontario (ID: 2024–019.01). It was also approved after a privacy impact assessment (ID: PHO.2024.5.6). The Canadian Health Survey on Children and Youth data are deidentified participant data, and informed consent was not required.

### Data source

The 2019 CHSCY is a cross-sectional survey collected from February 11, 2019, through August 2, 2019, to examine health data relevant to Canadian children and youth (Statistics Canada, 2024). The survey covers the Canadian population of children and youth dwelling within the ten provinces and the three territories between the ages of 1 and 17 as of January 31, 2019 (Statistics Canada, 2024). Children and youth living in First Nations reserves or other Aboriginal settlements, foster homes, and institutional settings were excluded. The Canadian Child Tax Benefit (CCB) was used as a sampling frame, which covers approximately 98% of the Canadian population aged 1 to 17 years in all provinces and 96% in all territories (Statistics Canada, 2024). The CHSCY incorporated electronic questionnaires that were either self-completed by respondents or completed with the assistance of an interviewer over the telephone. For children aged 1–11 years, questionnaires were administered to the person most knowledgeable (PMK) about the selected child. Participation in the survey was voluntary. Sampling was stratified by age (1–4, 5–11, and 12–17) and by geographical location (sub-local health integration networks, provinces, and territories) (Statistics Canada, 2024). This study used data from PMK questionnaires for children aged 1 to 11 years in Canada. The response rates were 58.9% for ages 1–4 years and 57.8% for ages 5–11 years (Statistics Canada, 2024). In the survey, the PMK was the birth parent in 97% of the responses. In this paper, PMK will be referred to as the “parent” of the child.

### Independent variable: positive parent–child interaction

Positive PCI was measured using five items in the CHSCY, which assessed interactions between the parent and child during the week. Items included the frequency with which the parent engaged in the following behaviours: (i) praised their child, (ii) focused attention on their child, (iii) laughed with their child, (iv) did something special with their child, and (v) played sports/hobbies/games with their child. Response categories for each of the five PCI questions were on a 5-point rating scale (i.e., “Never” = 0, “About once a week or less” = 1, “A few times a week” = 2, “One or two times a day” = 3, “Many times each day” = 4). Similar to previously published reports (Middlesex-London Health Unit & Windsor-Essex Health Unit, 2004; Northwestern Health Unit, 2019), the combined PCI score was calculated by summing responses across all five items, with a possible range from 0 to 20. Higher scores indicated higher levels of positive PCI.

### Primary analysis dependent variable: parent-reported general child mental health

The primary analysis outcome was general child mental health, as reported by the parent. In the questionnaire, the parent was asked the following question, “In general, how is this child’s mental health?”, with responses on a 5-point rating scale (i.e., “Excellent” = 1, “Very good” = 2, “Good” = 3, “Fair” = 4, “Poor” = 5). Due to a small number of responses in the “Fair” and “Poor” categories, this variable was dichotomized into good (excellent/very good/good) and poor (fair/poor) to assess the risk of reporting “fair/poor” general child mental health.

### Secondary analysis dependent variable: diagnosis with an anxiety or a mood disorder

A secondary analysis was conducted in children aged 5–11 years by creating a model using a survey question regarding whether children had ever received a mental health condition diagnosis from a health care professional. In the survey, each parent participant was asked: “Has this child been diagnosed with any of the following long-term conditions? 1) An anxiety disorder, such as a phobia, obsessive–compulsive disorder or a panic disorder 2) A mood disorder such as depression, bipolar disorder, mania or dysthymia”. These questions were only asked of parents of children aged 5 and older. Due to small numbers of respondents reporting these diagnoses, mood and anxiety disorders were combined, and this outcome was dichotomized to compare children who were diagnosed with an anxiety or a mood disorder to those who were not.

### Covariates

Confounders between the exposures and outcomes to include in the models were selected a priori. These were child age (years) (Shan et al., 2019), child sex at birth (male or female) (Shan et al., 2019), household income (which was adjusted for household size by dividing by the square root of the number of household members) (Katsantonis & McLellan, 2024; Shan et al., 2019; Statistics Canada, 2024), race and ethnic origin (White/non-racialized, Black, East Asian, Southeast Asian/Filipino, West Asian/Arab, South Asian, Latin American, Other/Multiple, and Indigenous identity) (Katsantonis & McLellan, 2024; Tolliver‐Lynn et al., 2021; Zarei et al., 2023), parental divorce/separation (yes or no) (Shan et al., 2019), highest level of education of parent (high school or less, college/trades, and Bachelor’s or more) (Shan et al., 2019), child immigration status (non-immigrant or immigrant/non-permanent resident) (Harris & Chen, 2023), parent self-perceived mental health (excellent/very good, good, and fair/poor) (Katsantonis & McLellan, 2024), and parent self-perceived life stress (not at all stressful/not very stressful, a bit stressful, and quite a bit stressful/extremely stressful) (Farmer & Lee, 2011).

### Statistical analysis

A series of descriptive statistics were used to summarize the distribution of PCI items and sociodemographic characteristics in the study sample. Continuous variables (i.e., child age, household income, and combined PCI score) were assessed for their normality. No variable followed a normal distribution; therefore, medians and interquartile range (IQR) were reported for continuous variables. Frequencies and 95% confidence intervals (CIs) were reported for categorical variables. The percentage of missing data for the PCI items was very low (range 0.26–0.66%). Therefore, missing data was treated as missing at random, and a complete case analysis was conducted to exclude participants with missing data.

Unadjusted associations between the combined PCI score and general child mental health, as well as between each of the confounding variables and general child mental health, were calculated using separate bivariate logistic regression models. These models generated odds ratios (ORs) and corresponding 95% CIs to be compared with estimates from the adjusted multivariable model. Multivariable logistic regression models, accounting for confounders, were performed to generate ORs and corresponding 95% CIs.

To assess the impact of the five PCI items on general child mental health, unadjusted separate bivariate logistic regression models and multivariable logistic regression models adjusted for confounders were conducted assessing the association between each individual PCI item and general child mental health. Due to low sample sizes, the lowest three response categories in the five PCI items were combined to report “a few times a week or less” category.

Analyses were conducted using SAS Enterprise Guide (V8.2). Proc Survey commands with bootstrap replication (*n* = 1000) and Statistics Canada’s bootstrap weights were applied to account for the complex survey design. Collinearity was assessed using the variance inflation factor. None of the variance inflation factors exceeded a value of 2, indicating no significant collinearity.

## Results

**Fig. 1:**
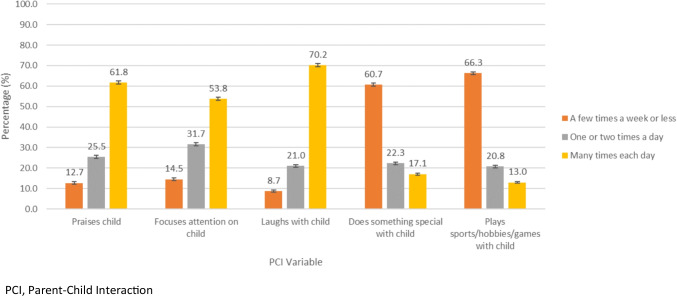
Distribution of how often parents interact with their child for each individual PCI item in children ages 1–11 years; Canada, 2019

A total of 28,874 Canadian children aged 1–11 years were included in this study. Characteristics of the study sample are summarized in Table 1. The median combined PCI score was 15.7 (IQR 13.8–17.5). Only 2.8% of parents reported their child’s mental health to be “fair/poor”, while 3.4% of parents reported an anxiety or a mood disorder diagnosis in children ages 5–11 years. Characteristics of the individual PCI items are summarized in Fig. 1. Most parents laughed with their child many times each day (70.2%), praised their child many times each day (61.8%), and focused their attention on their child many times each day (53.8%). Conversely, 17.1% of parents reported doing something special with their child many times each day, and only 13.0% reported playing sports/hobbies/games with their child many times each day.

**Table 1:** Study sample characteristics of children ages 1–11 years; Canada, 2019

Variable	Value^a^
Combined PCI (median [IQR])	15.7 (13.8–17.5)
Child age (median years [IQR])	5.5 (2.8–8.3)
Child sex at birth	
Female	48.7 (48.7, 48.7)
Male	51.3 (51.3, 51.3)
Household income adjusted for household size	
< $25,000	24.8 (24.1, 25.6)
$25,000 to 49,999	32.4 (31.6, 33.2)
$50,000 to 74,999	21.9 (21.3, 22.6)
$75,000 to 99,999	11.9 (11.4, 12.5)
$100,000 to 149,999	6.4 (6.0, 6.8)
$150,000 to 199,999	1.3 (1.1, 1.4)
$200,000 and higher	1.2 (1.0, 1.4)
Race and ethnic origin	
Black	5.7 (5.3, 6.1)
East Asian	4.8 (4.5, 5.1)
Indigenous Identity	5.1 (4.8, 5.5)
Latin American	1.1 (0.9, 1.3)
Other/Multiple	2.2 (2.0, 2.4)
South Asian	7.3 (6.9, 7.7)
Southeast Asian/Filipino	4.0 (3.7, 4.4)
West Asian/Arab	3.2 (2.8, 3.5)
White/non-racialized	66.6 (65.8, 67.3)
Parental divorce/separation	
Yes	14.9 (14.3, 15.5)
No	85.1 (84.5, 85.7)
Highest level of education of parent	
High school or less	20.1 (19.4, 20.7)
College/vocational/university certificate or diploma	37.5 (36.7, 38.4)
Bachelor’s or more	42.4 (41.6, 43.2)
Child immigration status	
Non-immigrant	93.3 (92.8, 93.7)
Immigrant/non-permanent resident	6.7 (6.3, 7.2)
Parent self-perceived mental health	
Excellent/very good	72.3 (71.6, 73.0)
Good	22.4 (21.7, 23.0)
Fair/poor	5.3 (5.0, 5.7)
Parent self-perceived life stress	
Not at all stressful/not very stressful	26.5 (25.8, 27.3)
A bit stressful	47.7 (46.9, 48.5)
Quite a bit stressful/extremely stressful	25.8 (25.1, 26.4)
Parent-reported general child mental health	
Excellent/very good/good	97.2 (97.0, 97.5)
Fair/Poor	2.8 (2.5, 3.0)
Diagnosis with an anxiety or a mood disorder in children ages 5–11	
Yes	3.4 (3.0, 3.8)
No	96.6 (96.2, 97.0)

*CI* confidence interval, *PCI* parent–child interaction, *IQR* interquartile range

^a^All values are percentages (95% CIs) unless otherwise noted

In the unadjusted model of the association between combined PCI score and parent-reported general child mental health (Table 2), each one-unit increase in combined PCI score was significantly associated with a 15% decrease in the odds of reporting “fair/poor” general child mental health (OR = 0.85; 95%CI 0.83–0.88). However, after adjusting for confounders, the OR was attenuated to 0.96 (95%CI 0.91–1.01) and was no longer statistically significant. In the multivariable model, significant associations remained for child age, child sex at birth, parental divorce/separation, parent self-perceived mental health, and parent self-perceived life stress (see Table 2). Notably, there was a strong association between “fair/poor” parent self-perceived mental health and the odds of reporting “fair/poor” general child mental health (OR = 12.18; 95%CI 8.88–16.71).

**Table 2:** Unadjusted and adjusted OR (95% CI) for parent-reported fair/poor mental health by PCI and all covariates in children ages 1–11 years in Canada

Variable	Unadjusted OR based on separate bivariate models (95% CI)	Adjusted OR based on multivariable model^a^ (95% CI)
Combined PCI (increase 1 unit)	0.85* (0.83–0.88)	0.96 (0.91–1.01)
Child age (years)	1.27* (1.22–1.31)	1.24* (1.19–1.29)
Child sex at birth		
Female	REF	REF
Male	1.74* (1.39–2.17)	1.94* (1.51–2.48)
Household income (adjusted for household size, per CAD$10,000)	0.93* (0.90–0.96)	0.97 (0.94–1.01)
Race and ethnic origin		
White/non-racialized	REF	REF
Black	0.72 (0.44–1.18)	0.92 (0.54–1.59)
East Asian	0.49* (0.30–0.80)	0.74 (0.44–1.26)
Southeast Asian/Filipino	0.40* (0.19–0.82)	0.64 (0.29–1.41)
West Asian/Arab	0.51 (0.15–1.73)	0.72 (0.18–2.97)
South Asian	0.65 (0.41–1.03)	1.44 (0.88–2.34)
Latin American	0.57 (0.14–2.36)	0.82 (0.19–3.57)
Other/multiple	0.75 (0.34–1.64)	0.74 (0.32–1.68)
Indigenous identity	2.09* (1.47–2.98)	1.49 (1.00–2.22)
Parental divorce/separation		
No	REF	REF
Yes	3.64* (2.92–4.54)	1.66* (1.27–2.16)
Highest level of education of parent		
High school or less	REF	REF
College/vocational/university certificate or diploma	0.83 (0.65–1.08)	0.94 (0.71–1.25)
Bachelor’s or more	0.50* (0.38–0.64)	0.74 (0.54–1.00)
Child immigration status		
Non-immigrant	REF	REF
Immigrant/non-permanent resident	0.54 (0.31–0.93)	0.65 (0.34–1.22)
Parent self-perceived mental health		
Excellent/very good	REF	REF
Good	5.09* (3.91–6.62)	3.53* (2.67–4.67)
Fair/poor	20.07* (15.21–26.49)	12.18* (8.88–16.71)
Parent self-perceived life stress		
Not at all stressful/not very stressful	REF	REF
A bit stressful	1.95* (1.32–2.89)	1.25 (0.83–1.88)
Quite a bit stressful/extremely stressful	6.67* (4.55–9.77)	2.66* (1.75–4.04)

*CI* confidence interval, *OR* odds ratio, *PCI* parent–child interaction

^a^Adjusted for child age, child sex at birth, household income, race and ethnic origin, parental divorce/separation, highest level of education of parent, child immigration status, parent self-perceived mental health, and parent self-perceived life stress

^*^Indicates statistical significance at *p* < 0.05

In the unadjusted models assessing the association between each PCI item and parent-reported general child mental health (Table 3), most PCI items displayed a dose response association, where increasing quantities of interaction corresponded with a decrease in odds of reporting “fair/poor” general child mental health. However, when the models were adjusted for confounders, almost all associations became non-significant, with only the “laughs with child” maintaining a significant association. Compared to parents who laughed with child “a few times a week or less”, parents who laughed with child “many times each day” had an OR of 0.64 (95%CI 0.47–0.89) or 36% decreased odds of reporting “fair/poor” general child mental health.

**Table 3:** Unadjusted and adjusted OR (95% CI) for parent-reported fair/poor mental health by individual PCI items in children ages 1–11 years in Canada

Variable	Unadjusted OR based on separate bivariate models (95% CI)	Adjusted OR based on multivariable model^a^ (95% CI)
PCI – praises child		
A few times a week or less	REF	REF
One or two times a day	0.76 (0.55–1.05)	0.98 (0.69–1.39)
Many times each day	0.61* (0.46–0.80)	1.14 (0.81–1.62)
PCI – focuses attention on child		
A few times a week or less	REF	REF
One or two times a day	0.81 (0.63–1.06)	1.08 (0.81–1.45)
Many times each day	0.45* (0.35–0.59)	0.96 (0.70–1.32)
PCI – laughs with child		
A few times a week or less	REF	REF
One or two times a day	0.50* (0.38–0.67)	0.79 (0.57–1.09)
Many times each day	0.30* (0.23–0.39)	0.64* (0.47–0.89)
PCI – does something special with child		
A few times a week or less	REF	REF
One or two times a day	0.61* (0.45–0.82)	1.12 (0.81–1.54)
Many times each day	0.34* (0.25–0.48)	0.90 (0.62–1.32)
PCI – plays sports/hobbies/games with child		
A few times a week or less	REF	REF
One or two times a day	0.60* (0.44–0.83)	1.21 (0.84–1.74)
Many times each day	0.31* (0.20–0.49)	0.83 (0.51–1.36)

*CI* confidence interval, *OR* odds ratio, *PCI* parent–child interaction

^a^Adjusted for child age, child sex at birth, household income, race and ethnic origin, parental divorce/separation, highest level of education of parent, child immigration status, parent self-perceived mental health, and parent self-perceived life stress

^*^Indicates statistical significance at *p* < 0.05

In the secondary analysis examining anxiety or mood disorder diagnoses, we found similar results to our primary analyses (Table 4). There was a significant association between combined PCI score and having an anxiety or a mood disorder diagnosis in the unadjusted model (OR = 0.86; 95%CI 0.84–0.88). However, this association was not significant after adjusting for confounders (OR = 1.01; 95%CI 0.98–1.05). In the multivariable model, significant associations remained for child age, child sex at birth, household income, race and ethnic origin, parental divorce/separation, highest level of education of parent, child immigration status, parent self-perceived mental health, and parent self-perceived life stress (see Table 4). Similar to the primary analysis, there was a strong association between “fair/poor” parent self-perceived mental health and the odds of reporting “fair/poor” general child mental health (OR = 3.90; 95%CI 2.63–5.79).

**Table 4:** Unadjusted and adjusted OR (95% CI) for diagnosis with an anxiety or a mood disorder by PCI and all covariates in children ages 5–11 years in Canada

Variable	Unadjusted OR based on separate bivariate models (95% CI)	Adjusted OR based on multivariable model^a^ (95% CI)
Combined PCI (increase 1 unit)	0.95* (0.90–0.99)	1.04 (0.99–1.10)
Child age (years)	1.29* (1.21–1.38)	1.31* (1.22–1.41)
Child sex at birth		
Female	REF	REF
Male	1.56* (1.20–2.02)	1.57* (1.20–2.05)
Household income (adjusted for household size, per CAD$10,000)	0.97 (0.94–1.00)	0.98 (0.94–1.02)
Race and ethnic origin		
White/non-racialized	REF	REF
Black	0.49 (0.24–1.01)	0.62 (0.29–1.30)
East Asian	0.58 (0.30–1.12)	0.80 (0.40–1.61)
Southeast Asian/Filipino	0.28* (0.11–0.70)	0.41 (0.16–1.06)
West Asian/Arab	0.20* (0.05–0.72)	0.25* (0.07–0.95)
South Asian	0.25* (0.09–0.71)	0.42 (0.15–1.18)
Latin American	0.64 (0.07–5.67)	0.80 (0.09–6.99)
Other/multiple	1.00 (0.33–3.01)	1.12 (0.38–3.34)
Indigenous identity	1.70* (1.09–2.66)	1.25 (0.77–2.03)
Parental divorce/separation		
No	REF	REF
Yes	2.14* (1.63–2.8)	1.25 (0.92–1.70)
Highest level of education of parent		
High School or less	REF	REF
College/vocational/university certificate or diploma	1.18 (0.86–1.63)	1.30 (0.93–1.82)
Bachelor’s or more	0.74 (0.53–1.03)	1.04 (0.72–1.50)
Child immigration status		
Non-immigrant	REF	REF
Immigrant/non-permanent resident	0.38* (0.20–0.72)	0.71 (0.36–1.43)
Parent self-perceived mental health		
Excellent/very good	REF	REF
Good	3.44* (2.58–4.58)	2.59* (1.90–3.54)
Fair/poor	5.93* (4.18–8.41)	3.90* (2.63–5.79)
Parent self-perceived life stress		
Not at all stressful/not very stressful	REF	REF
A bit stressful	1.90* (1.31–2.77)	1.42 (0.96–2.08)
Quite a bit stressful/extremely stressful	4.88* (3.40–7.00)	2.78* (1.88–4.12)

*CI* confidence interval, *OR* odds ratio, *PCI* parent–child interaction

^a^Adjusted for child age, child sex at birth, household income, race and ethnic origin, parental divorce/separation, highest level of education of parent, child immigration status, parent self-perceived mental health, and parent self-perceived life stress

^*^Indicates statistical significance at *p* < 0.05

## Discussion

This study explored the association between positive PCI and general child mental health in children ages 1–11 years in Canada using a nationally representative survey. To our knowledge, this is the first population-based study to assess the association between positive PCI and child health in Canada based on the five PCI items used in the CHSCY survey. In the unadjusted models, combined PCI score and all individual PCI items displayed a significant association with general child mental health as reported by the parents. However, after adjusting for confounders, the only remaining significant association was between “laughs with child” and general child mental health. Similarly, in the secondary analysis assessing an anxiety or a mood disorder diagnosis in children ages 5–11, significant associations were found in the unadjusted model between combined PCI score and anxiety or mood disorder diagnosis, which were attenuated in the adjusted model.

Our findings did not align with some population-based studies; however, it is important to note that these studies used different measures for PCI, and their measures had been validated. In Shan et al. (2019), the CPCIS was a 12-item scale with individual items ranging from 1 to 5. This scale measured more individual-level interactions between the parent and the child (e.g., how often the parent draws pictures with their child, how often the parent teaches concepts to their child). Similarly, the Pianta scale used by Katsantonis and McLellan (2024) was a 30-item scale with individual items ranging from 1 to 5. This scale closely measured how parents feel about multiple dimensions of their relationships with the child (e.g., does the child openly share their feelings and experiences with the parent?). Contrary to Shan et al. (2019) and Katsantonis and McLellan (2024), our study assessed positive PCI using five items that may not always reflect positive interaction. For example, while our construct included how often parents focus attention on the child, some studies have shown that too much parental involvement may have adverse effects (e.g., anxiety disorders and poor self-regulation) (Hudson & Rapee, 2001; Obradović et al., 2021). For instance, one study of 95 children between the ages of 7 and 15 years in Australia found an association between parental over-involvement and child anxiety disorders (Hudson & Rapee, 2001). The mechanism by which this may occur is that parents who are overinvolved in their child’s activities may reduce their child’s sense of control over events. Moreover, in contrast to the CPCIS, our construct did not account for cultural factors. Many elements of parenting, such as caregiving and warmth, are culturally dependent. For example, in some cultures, parents tend to display warmth by showing affection (e.g., hugging the child), while in other cultures they may display warmth by taking care of the child’s education (Lansford, 2022). While our study showed mostly non-significant associations, the results may be due to the quality of this specific population-level indicator of PCI and not representative of the true association of PCI and mental health.

There was no consistent dose–response or clear pattern when examining the adjusted associations between each PCI item individually and general child mental health. These findings differ from the well-established literature on the relationship between positive parenting practices and child mental health issues (Ge et al., 2009). Positive parenting practices, including social support, serve as protective factors for stressful life events and directly enhance mental health (Ge et al., 2009). The lack of consistent findings may be because the PCI measures used in the CHSCY may not have construct validity in capturing PCI variables relevant to predict child mental health outcomes.

In the adjusted model, we found that laughing with their child “many times each day” was associated with reduced risk of “fair/poor” mental health compared to laughing “a few times a week or less” highlighting the positive impact of laughing with child in the early years and mental health outcomes. This is consistent with longitudinal studies which have shown that high frequency of laughter has a positive effect on stress and cardiovascular health (Oliveira & Arriaga, 2022; Zander-Schellenberg et al., 2020). This finding may be explained in that higher frequency of laughter reduces the release of stress hormones within the body and encourages the release of endorphins that improve mood (Yim, 2016). On the other hand, frequent laughter with the child could signify other PCI indicators that were not captured in our analysis, such as having more time to share affection or delight the child.

In our study, we found strong associations between parents’ self-perceived mental health and the mental health of their children in the unadjusted and adjusted models. This is consistent with evidence in the literature, including a systematic review of 83 studies by Stracke et al. (2023), which showed an association between parental mental health and child mental health outcomes across 25 meta-analyses. Moreover, Stracke et al. (2023) identified that dysfunctional PCI may play a role in the transmission of mental disorders from parents to their children by mediating the association. This finding is similar to a study by Low and Stocker (2005) in the United States, which found that parental mental issues, including depression, can limit positive PCI (e.g., reading to child) which in turn impacts the child’s mental development. As the mediation analysis of positive PCI was beyond the scope of this study, future studies should explore the role positive PCI plays in mediating the association between parental mental health issues and child outcomes.

Some of the strengths of this study include the use of CHSCY data, which is nationally representative of the Canadian population. The large, representative sample suggests that the findings have high generalizability within this age range. Moreover, this is the first study to examine the PCI items used in the CHSCY to investigate empirical evidence of their association with an important child health outcome in children 1–11 years in Canada.

There are several limitations that should be considered when interpreting the results of this study. Firstly, the cross-sectional nature of the survey does not allow for causal inferences to be made. Secondly, the response rate in the CHSCY for ages 1–11 years was low (Statistics Canada, 2024). However, bootstrap replication and weights provided by Statistics Canada were applied, which attempted to mitigate the impact of the low response rate. Additionally, the PCI items in the CHSCY have not been tested for construct validity. These items were also reported by the parents, which means they are likely to be influenced by multiple biases as parents may overestimate their interactions with their children due to factors such as social pressure and desirability (Hofferth, 2006). Similarly, the reliance on parents to report on child mental health may not truly reflect the general mental health status of the child (Caqueo-Urízar et al., 2022). Since these reporting biases are likely random, they may bias the observed effect towards the null, leading to an underestimation of the true association. Moreover, this outcome was dichotomized to account for low sample sizes, which risks oversimplifying and misinterpreting the complex relationship between PCI and child mental health outcomes. These issues may have been addressed in that we conducted a secondary analysis using an outcome that captured whether children ages 5–11 had ever received a diagnosis for an anxiety or a mood disorder. As this analysis found similar results, it lends some confidence to our findings. However, both the primary and secondary outcomes, as well as some covariates, had a low prevalence (e.g., 2.8% for fair/poor child mental health). Given these low frequencies, the models may have experienced a loss of precision, likely reducing the power to detect associations. Lastly, the combined PCI score for the sample was generally high and left skewed. Therefore, it is unclear if assessing one-unit increases in the score is a clinically meaningful estimate.

## Conclusion

This study did not find a consistent association between PCI and mental health outcomes in children ages 1–11 years in Canada using the 2019 CHSCY, except for the frequency at which the parent laughs with the child. This suggests that PCI, used as a total score of the five items in the CHSCY, is not a strong indicator of child mental health and therefore may not provide a good indication of parenting for public health assessment and surveillance activities. The “laughs with child” item independently may be a more promising indicator of child mental health outcomes. Yet, as PCI is an important parenting concept, the PCI items in the CHSCY may not adequately capture the intended construct. Future studies should consider assessing the construct validity of the PCI items used in the CHSCY.

## Contributions to knowledge

What does this study add to existing knowledge?

- The prevalence of mental health disorders in Canada has increased over the past 10 years. Positive parent–child interaction (PCI) is a potential protective factor for child mental health, but has not been explored in a Canadian context, especially at a population level.
- This study assessed the association between positive PCI and mental health outcomes in children using a nationally representative sample of children ages 1–11 and did not find a consistent association between PCI and child mental health, as reported by the parent.

What are the key implications for public health interventions, practice, or policy?

- Measuring parenting has important implications in guiding policy and program recommendations for positive parenting practices. However, measuring parenting at the population level presents many challenges, including multiple contextual and cultural considerations.
- The results of this study reveal that the PCI items used in the CHSCY may not adequately capture the intended parenting construct and therefore may not provide a good indication of parenting for public health assessment and surveillance activities.

## Data Availability

Data may be obtained from a third party and are not publicly available. The Canadian Health Survey on Children and Youth data are deidentified participant data, available on successful application to Statistics Canada’s Research Data Centre programme: https://www.statcan.gc.ca/eng/rdc/index.

## Code availability

Analyses were conducted using SAS Enterprise Guide (V8.2).
